# Recurrence of emphysema post-lung transplantation in a patient with alpha 1 antitrypsin deficiency (AATD)

**DOI:** 10.1016/j.rmcr.2020.101309

**Published:** 2020-11-26

**Authors:** Ali Ataya

**Affiliations:** Division of Pulmonary, Critical Care and Sleep Medicine, University of Florida Health, Gainesville, FL, 32610-0225, USA

**Keywords:** Alpha 1 antitrypsin deficiency, Alpha 1 antitrypsin therapy, Chronic obstructive pulmonary disease, Emphysema, Lung transplantation, Smoking, AAT, alpha 1 antitrypsin, AATD, alpha 1 antitrypsin deficiency, COPD, chronic obstructive pulmonary disease, CT, computed tomography, FEV_1_, forced expiratory volume in 1 s, FVC, forced vital capacity

## Abstract

The genetic disorder alpha 1 antitrypsin deficiency (AATD) results in reduced levels of alpha 1 antitrypsin (AAT) in the lung and an imbalance between AAT anti-protease activity and the activity of proteases that degrade elastin and connective tissues. This imbalance commonly leads to the excessive proteolysis of structural tissue of the alveoli, causing chronic obstructive pulmonary disease (COPD)/emphysema. While patients with AATD are encouraged to make lifestyle changes, including stopping smoking, and can be treated with alpha 1 antitrypsin therapy (AAT therapy) to slow progression of COPD/emphysema, damage to the lungs is irreparable, and therefore, lung transplantation is required in severe cases. However, following lung transplant, the genetic cause of AATD-related COPD/emphysema remains, and patients may continue to be at risk of redeveloping COPD/emphysema.

Recurrence of COPD/emphysema was observed in a patient with AATD 2 years after initial successful lung transplantation and cessation of AAT therapy who recommenced smoking after no signs of disease at the 1-year assessment. This case demonstrates that smoking cessation is critical in patients with AATD, even after lung transplant, and it highlights that patients with AATD may benefit from AAT therapy post-lung transplant.

## Highlights

• Patients with AATD can redevelop emphysema following lung transplantation.
• Abstinence from smoking remains crucial in all patients with AATD.
• AAT therapy may be required in patients who redevelop emphysema post-lung transplant.

## 1 Introduction

In 2016, the Registry of the International Society for Heart and Lung Transplantation reported that the most common primary indication for lung transplantation worldwide was COPD. Approximately one-third of all lung transplants globally were COPD-related and approximately 5% of these cases were reported to be in patients with AATD [1].

Patients with AATD can reduce the progression of emphysema by ensuring lifestyle changes, such as smoking cessation, are implemented and maintained following diagnosis [2,3]. In some patients with severe AATD-related emphysema, lung transplantation is required, and is associated with a 5-year survival rate of approximately 60% [4]. However, one of the main issues in patients with AATD-related vs. non-genetic COPD-related emphysema requiring lung transplantation is that transplantation does not resolve the underlying genetic deficiency that originally led to the development of COPD/emphysema. As a result, following transplantation, patients remain at risk of redeveloping emphysema and the related clinical sequelae, e.g., exacerbations of COPD; it is therefore crucial that lifestyle changes are sustained [4].

## 2 Case description

The patient is a 59-year-old male with severe AATD who was diagnosed with the PI*ZZ genotype in 2004 at the age of 44. His medical history includes arterial hypertension and type-2 diabetes mellitus. He is an ex-smoker who consumes a moderate amount of alcohol (2 units/week). Patient characteristics are reported in Table 1.

**Table 1:** Patient characteristics.

Age (years) at diagnosis	44
Age (years) at data collection^a^	59
Race	Caucasian
Height (m)	1.82
Weight (kg)	85.9
BMI (kg/m^2^)	25.9
Smoking status	Ex-smoker (10 pack years)

BMI, body mass index.

a Data were collected in 2019.

At diagnosis, the patient's alpha 1 antitrypsin (AAT) level was 36 mg/dL, and his forced expiratory volume in 1 s (FEV_1_) and forced vital capacity (FVC) were 0.85 L (23% predicted) and 2.8 L (55% predicted), respectively. After diagnosis, he stopped smoking, he was prescribed AAT therapy (Prolastin-C®) at the standard dose of 60 mg/kg bodyweight/week.

**Fig. 1:**
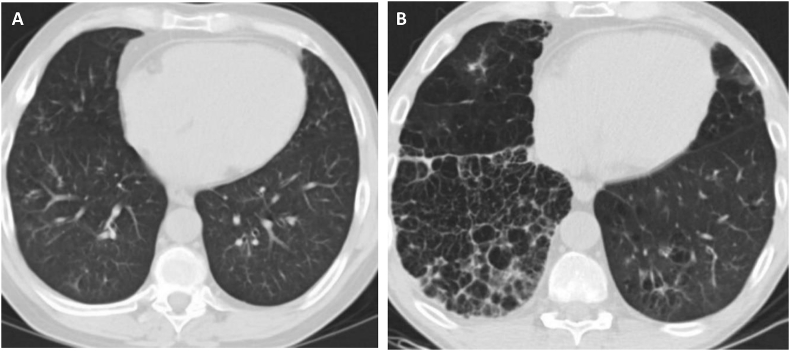
Chest CT scan with A) normal lung parenchyma one year following bilateral lung transplantation and B) bibasilar predominant emphysema (right greater than left), with pleural inflammation associated with chest tube placement, two years post-transplant CT, computed tomography.

In 2010, the patient underwent a bilateral lung transplantation as a result of advanced emphysema. Following the successful transplantation, AAT therapy was stopped; there were no signs of graft rejection on lung biopsy and no evidence of infections or complications. At the patient's 1-year follow up, his FEV_1_ and FVC were 2.31 L (63% predicted) and 5.21 L (103% predicted), respectively. There was concern that the patient had developed bronchiolitis obliterans syndrome (BOS) and he was treated with a tapering dose of prednisone, and his immunosuppressant treatment was altered by the transplant team. However, a transbronchial lung biopsy did not show any evidence of rejection. A chest computed tomography (CT) scan was performed and no lung pathologies (including emphysema) were identified (Fig. 1A).

The patient subsequently re-commenced cigarette smoking, a few cigarettes (less than one pack) a day, for roughly one year. Approximately 2 years post-transplant he presented with a right-sided large-volume pneumothorax, which required chest tube placement. At this time, chest CT scan revealed signs of basally predominant emphysema in the transplanted lungs (Fig. 1B), and his clinical assessments were FEV_1_ 1.9 L (46% predicted) and FVC 4.29 L (80% predicted). The patient likely had a superimposed consolidation in the right lower lobe, and due to recent insertion of chest tube, there was associated pleural inflammation and small right sided pleural effusion (Fig. 1B). No other risk factors, besides smoking, were observed ahead of presentation.

The patient then quit smoking and recommenced his pre-transplant AAT therapy regimen. In 2014, the patient's AAT level was 97 mg/dL, and he has now been treated with AAT therapy for more than 5 years. In this time, he had an average of one moderate exacerbation (requiring oral antibiotics and/or corticosteroids) per year. At his most recent clinical assessment in August 2019, his FEV_1_ and FVC were 2.2 L (55% predicted) and 5.1 L (100% predicted) respectively, with an oxygen saturation of 96%.

## 3 Discussion

The recurrence of emphysema following lung transplantation demonstrates that the underlying systemic condition is not resolved in AATD-related lung transplant cases. It is well known that smoking accelerates the progression of lung disease in patients with AATD [5] as well as in individuals who have undergone lung transplant [6]; therefore, it is crucial that patients with AATD abstain from smoking after diagnosis, even following lung transplantation. One other case of emphysema recurrence following AATD-related lung transplantation has been documented in the literature, and similar to the present case, it was reported to be related to the patient taking up smoking again [7].

In this case, the patient was treated with AAT therapy after the recurrence of emphysema post-transplant; however, there may be a rationale for recommencing AAT therapy for a short period following transplantation. Reports have indicated that treatment with AAT after transplantation may be beneficial due to the immunomodulatory and anti-inflammatory effect of AAT, may counter increased neutrophil activity, and re-establish protease-antiprotease balance [8,9]. However, treatment with AAT post-transplant is currently outside of licensed indications (AAT therapy is indicated for treatment of emphysema). In addition, other factors might be considered. For example, treatment with intravenous injections in immunocompromised patients may increase the risk of infections or complications. Plus, there are the factors generally associated with AAT therapy, such as quality of life for patients requiring frequent injections, potential for development of antibodies, and the cost of therapy [10,11]. Therefore, further studies on treatment with AAT therapy after lung transplantation are required.

## 4 Conclusions and implications

Following lung transplantation, this patient with AATD developed progressive emphysema in a setting of active smoking; his lung function deterioration was slowed by smoking cessation and re-initiation of AAT therapy. This emphasizes the importance of smoking cessation following a diagnosis of AATD, including after lung transplantation. Furthermore, the outcomes of this case suggest that treatment with AAT therapy post-transplant in patients with AATD may be beneficial in selected cases. There is a need for greater awareness of the consequences of smoking in patients with AATD and increased education on the risk factors for emphysema development, as well as further research on the effects of AAT therapy following lung transplantation.

## Funding

Medical writing support for this manuscript was funded by 10.13039/100008322CSL Behring. The sponsor had no role in the collection, analysis and interpretation of data; in the writing of the manuscript; and in the decision to submit the manuscript for publication.

## Declarations of competing interest

The author has no competing interests to declare.
